# Engineering Cu/NiCu LDH Heterostructure Nanosheet Arrays for Highly-Efficient Water Oxidation

**DOI:** 10.3390/ma16093372

**Published:** 2023-04-25

**Authors:** Ao-Bing Wang, Xin Zhang, Hui-Juan Xu, Li-Jun Gao, Li Li, Rui Cao, Qiu-Yan Hao

**Affiliations:** 1 Hebei Key Laboratory of Man-Machine Environmental Thermal Control Technology and Equipment, Hebei Vocational University of Technology and Engineering, Xingtai 054000, China; wab_509@163.com (A.-B.W.); zx_xtpc@163.com (X.Z.); gaoli_jun@foxmail.com (L.-J.G.); liliall@163.com (L.L.); hdcaorui@163.com (R.C.); 2 School of Materials Science and Engineering, Hebei University of Technology, Tianjin 300130, China

**Keywords:** Cu/NiCu LDH, heterostructure, water oxidation, density functional theory

## Abstract

The development of stable and efficient electrocatalysts for oxygen evolution reaction is of great significance for electro-catalytic water splitting. Bimetallic layered double hydroxides (LDHs) are promising OER catalysts, in which NiCu LDH has excellent stability compared with the most robust NiFe LDH, but the OER activity is not satisfactory. Here, we designed a NiCu LDH heterostructure electrocatalyst (Cu/NiCu LDH) modified by Cu nanoparticles which has excellent activity and stability. The Cu/NiCu LDH electrocatalyst only needs a low over-potential of 206 mV and a low Tafel slope of 86.9 mV dec^−1^ at a current density of 10 mA cm^−2^ and maintains for 70 h at a high current density of 100 mA cm^–2^ in 1M KOH. X-ray photoelectron spectroscopy (XPS) showed that there was a strong electronic interaction between Cu nanoparticles and NiCu LDH. Density functional theory (DFT) calculations show that the electronic coupling between Cu nanoparticles and NiCu LDH can effectively improve the intrinsic OER activity by optimizing the conductivity and the adsorption energy of oxygen-containing intermediates.

## 1. Introduction

Hydrogen energy is a clean energy with zero carbon emissions, and its high-quality energy density makes it the best choice to replace traditional fossil energy [1,2,3]. Electrochemical water splitting provides a reliable and environmentally friendly method for hydrogen production and can be combined with semiconductor light absorbing photoelectrodes, electrolytes and separation membranes to form photoelectrochemical (PEC) cells for solar-to-hydrogen (STH) energy conversion device [4,5,6,7,8]. However, the kinetics of oxygen evolution reaction (OER) in water electrolysis is slow, resulting in an increase in energy consumption of electrolytic water. Efficient electrocatalysts are needed to meet the needs of industrial production. Noble metal catalysts IrO_2_ and RuO_2_ can effectively reduce the reaction overpotential and are generally considered as one of the best catalysts for OER, but their scarcity and high cost greatly hinder their wide application [6,7,8]. Therefore, we have to focus on developing abundant, low-cost and high-efficiency non-noble metal OER electrocatalysts [9,10].

In recent years, many transition metal rich OER catalysts, such as 3D transition metal layered double hydroxides (LDH) [11,12], spinel oxides [13,14], selenide [8,14,15], sulfide [16,17], phosphide [18,19], etc., are expected to be used as highly active electrocatalysts for oxygen evolution reaction (OER). Recently, LDH based on 3D transition metals (especially Fe, Co, Mn, Ni) have been reported to exhibit excellent OER catalytic performance. In particular, Ni-based LDH has been the subject of in-depth research in recent years due to their abundance, corrosion resistance, and good flexibility of Ni [20,21,22]. The activity of iron-doped nickel-based LDH (NiFe LDH) is considered to be the most advanced OER catalyst in basic solutions. However, during the electrochemical test, the activity of NiFe LDH was severely reduced. Lin et al. [23] pointed out that in the OER process, Fe is segregated in the NiFe hydroxide lattice to form FeOOH. The dynamic metal dissolution segregation process accelerates the formation of the second phase FeOOH, resulting in the inactivation of the electrocatalyst. Markovic [24] studies have shown that although FeO_x_H_y_ is highly active, its potential-dependent dissolution rate at OER potentials is also high. Due to the high dissolution rate of Fe, the OER activity of FeO_x_H_y_ decreased significantly. Therefore, it is particularly important to explore iron-free LDHs with high activity and good stability. Due to the absence of iron, NiCu LDH has better stability than NiFe LDH, but is less active [25]. Researchers have made many efforts to improve the activity of nickel-based LDH, including heteroatom doping, heterostructure construction, and defect condition nickel based on its intrinsic activity. Among them, the construction of heterostructures proved to be an effective method since it had the advantage of improving the conductivity of the catalyst. The local electric field generated at the coupling interface of the heterostructure can effectively promote the dissociation of H_2_O at the LDH active site and adjust the electronic structure of the LDH active site to optimize the adsorption free energy of the reaction intermediate during the OER process [26]. Cu has a high work function (4.65) and good conductivity (conductivity 57,142,857 S/m) [27]. It is reasonable to speculate that the interface coupling of Cu and NiCu LDH (Cu/NiCu LDH) can not only effectively improve the conductivity of NiCu LDH but also cause strong electron interaction at their interface, which may improve the OER performance of NiCu LDH [28,29].

Herein, NiCu LDH were prepared by hydrothermal method on CC, and then Cu/NiCu LDH were prepared by chemical reduction method using copper acetate as Cu source. Considering that NiCu LDH has poor activity due to its adverse adsorption on oxygen-containing intermediates, our pioneering introduction of third-party nano-Cu particles to form a heterogeneous interface with NiCu LDH, which no other work has studied before, regulates the electronic structure of NiCu LDH material and can improve the conductivity of LDH and adjust the adsorption energy of active sites on oxygen containing intermediates, thereby optimizing the OER activity of the catalyst while ensuring electrocatalytic stability.

## 2. Materials and Methods

### 2.1. Material and Reagents

Ammonium fluoride (NH_4_F), urea (CO(NH_2_)_2_), nickel nitrate hexahydrate (Ni(NO_3_)_2_·6H_2_O), copper nitrate trihydrate (Cu(NO_3_)_2_·3H_2_O), copper acetate (Cu(Ac)_2_·H_2_O), sodium citrate (Na_3_C_6_H_5_O_7_·5H_2_O), and sodium borohydride (NaBH_4_) were used. All aqueous solutions were prepared with deionized water throughout the experiment. All chemicals are analytical grade and are not purified when used.

### 2.2. Synthesis of the Cu-NiCu and NiCu LDH Nanosheets

NiCu LDH was prepared by simple hydrothermal method at room temperature. First, 4 mmol NH_4_F, 10 mmol CO(NH_2_)_2_, 2 mmol Ni(NO_3_)_2_·6H_2_O and 2 mmol Cu(NO_3_)_2_·3H_2_O were dissolved in 55 mL deionized water. After stirring evenly under the action of magnetic stirrer, the prepared solution is transferred to 20 mL and 35 mL lined PTFE stainless steel autoclave. Put one carbon cloth (CC) into the 20 mL autoclave and two CC into the 35 mL autoclave crosswise to prevent the CC with one side from not absorbing particles. Seal the autoclave horizontally to prevent the relative position of CC from changing. The reaction temperature was set at 120 °C and the time was set to 6 h. After the reaction, the CC was rinsed forward and backward with deionized water, and finally dried in a vacuum oven at 80 °C.

Cu/NiCu LDH nanomaterials with different Cu loadings were prepared. First, 5 mmol Cu(Ac)_2_·H_2_O and 0.1176 g Na_3_C_6_H_5_O_7_·5H_2_O were dissolved in 40 mL deionized water. After being stirred evenly under the action of a magnetic stirrer, the dried NiCu LDH was put into a beaker and stirred slowly for 5 min. Then add 0.015 g NaBH_4_ and continue to stir slowly for 30 min. After stirring, the CC was washed forward and backward with deionized water and finally dried in a vacuum oven at 80 °C. Cu/NiCu LDH nanomaterials with different Cu loadings were synthesized by the same method by adding Cu(Ac)_2_·H_2_O of 2.5 and 1.25 mmol, respectively.

### 2.3. Characterization

The microstructure of the sample was characterized by scanning electron microscopy (SEM). X-ray diffraction (XRD) was carried out with Cu-Kα (λ = 0.15418 nm) as the target source with an X-ray diffractometer, and X-ray diffraction (XRD) was carried out in the range of 2θ = 10~90° at a scanning rate of 8° min^−1^. The elemental composition and surface chemical state of Cu/NiCu LDH and NiCu LDH were studied by X-ray photoelectron spectroscopy (XPS), and the electron interaction between Cu and NiCu LDH was studied.

### 2.4. Electrochemical Measurement Method

In a CHI604E electrochemical workstation, a three-electrode system was used for all electrochemical experiments. A graphite rod electrode served as the working electrode, a saturated calomel electrode (SCE) served as the counter electrode, and the catalyst placed on NF served as the reference electrode. Using the formula E (vs. RHE) = E (vs. Hg/HgO) + 0.924 V in 1 M KOH, the potentials recorded versus the saturated calomel electrode were calibrated to the reversible hydrogen electrode (RHE). At a scan rate of 2 mV/s, the linear sweep voltammetry (LSV) curves and Tafel curves were produced. Over a frequency range of 100 KHz to 0.01 Hz, electrochemical impedance spectroscopy (EIS) observations were made at 0.45926V with a 5 mV AC dither. By using scan rates of 5–100 mV s^−1^ during cyclic voltammetry testing in a non–Faradaic area, the double-layer capacitance (C_dl_) of the electrocatalyst was estimated.

### 2.5. DFT Calculation

Using the Projected Augmented Wave (PAW) technique, all density functional theory (DFT) computations were performed using the Vienna Ab-initio Simulation Package (VASP). The exchange and correlation effects were handled using the refined Perdew-Burke-Ernzerhof (PBE) functional, which has been proven to be successful. To model the catalytic contact, the (110) surface of Cu/NiCuOOH and NiCuOOH with 15 Å vacuum was used. For the purpose of geometry optimization of the slab surfaces, the Brillioun zone K points meshing was set up as a 3 × 3 × 1 grid centered at the gamma point. The cutoff energy is set to be 450 eV. The heterostructure Cu/NiCuOOH is created by connecting Cu-(101) and NiCuOOH-(110), with the mean strain to both surfaces of about 1%. The OER procedure consists of the next four stages:

*+OH^−^→ *OH + e^−^ (1)

*OH + OH^−^→*O + H_2_O+ e^−^ (2)

*O + OH^−^→*OOH + e^−^ (3)

*OOH + OH^−^→*+ O_2_ + H_2_O+ e^−^ (4)

Calculate the Gibbs free energy of each reaction intermediate using the calculated hydrogen electrode (CHE) model proposed by Nørskov et al. The free energy change (ΔG) of the OER intermediates (*OH, *O, and *OOH) was calculated by the following equation:

ΔG = ΔE + ΔZPE − TΔS + ΔG_U_ (5)

ΔE, ΔZPE, and ΔS are the adsorption energies of intermediates, the zero-point vibrational energy change calculated by the vibration frequency of adsorbates, and the entropy change between adsorbed and molecules states, respectively. In the calculation of ΔZPE, we fixed the catalyst atoms and only calculated the adsorption intermediates. For the entropy term, ΔS is the change in entropy (entropies of gas molecules were taken from standard values) and T was set to room temperature. The Gibbs free energy affected by the potential is expressed as ΔG_U_ = −neU, where n is the number of transferred electrons and U is the electrode potential relative to the standard hydrogen electrode.

Among them, ΔE is calculated by:

ΔE(*OH) = E(*OH) + 0.5 × E(H_2_) − E(H_2_O) − E(substrate) (6)

ΔE(*O) = E(*O) + E(H_2_) − E(H_2_O) – E(substrate) (7)

ΔE(*OOH) = E(*OOH) + 1.5 × E (H_2_) – 2 × E(H_2_O) − E(substrate) (8)

where E(*OH), E(*O), E(*OOH), E(substrate), E(H_2_), and E(H_2_O) represent the energies of adsorbed *OH, *O, *OOH, the substrate model, H_2_ and H_2_O molecules, respectively.

## 3. Results

**Figure 1:**
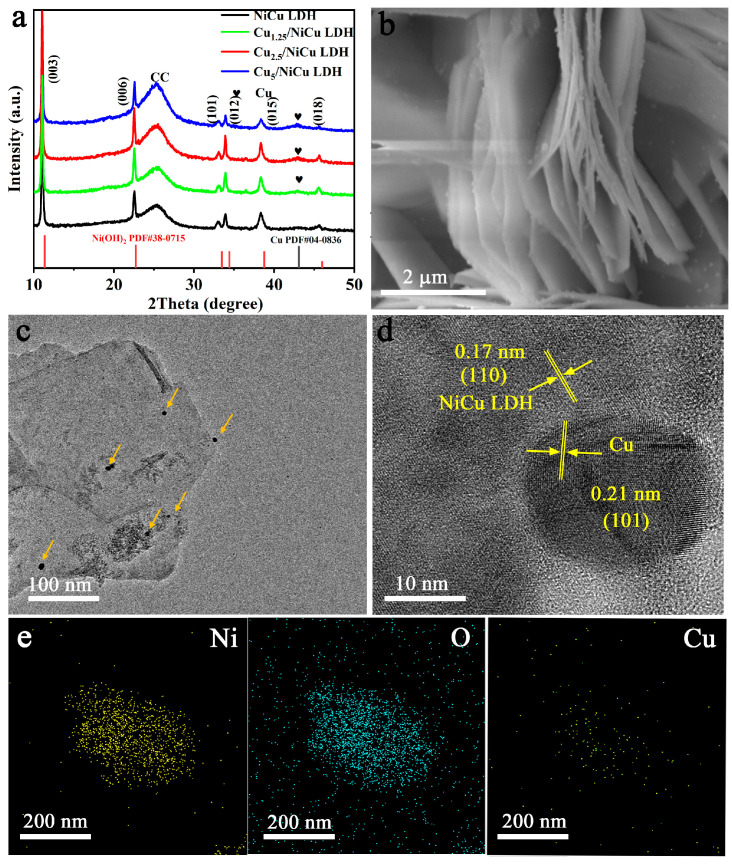
(**a**) XRD patterns of NiCu LDH, Cu_1.25_/NiCu LDH, Cu_2.5_/NiCu LDH and Cu_5_/NiCu LDH (Black heart: Cu). (**b**) SEM image of Cu_1.25_/NiCu LDH. (**c**) TEM, (**d**) HRTEM images of Cu_1.25_/NiCu LDH. (**e**) Element mapping of Cu_1.25_/NiCu LDH.

Cu/NiCu LDHs were prepared by hydrothermal and chemical reduction methods, as shown in Figure S1. First, NiCu LDH nanosheet arrays were synthesized on CC by a simple hydrothermal reaction. The Cu nanoparticles are then loaded onto NiCu LDH by chemical reduction. Cu/NiCu LDH with different Cu loading amounts was prepared by changing the input amount of the copper source, and was labeled Cu_1.25_/NiCu LDH, Cu_2.5_/NiCu LDH and Cu_5_/NiCu LDH, respectively. X-ray diffraction (XRD) is examined to confirm the presence of Cu nanoparticles and NiCu LDH phases [30]. As shown in Figure 1a, the characteristic peaks appearing at 11.3°, 22.7°, 35.4°, 34.4°, 38.7°, and 45.9° in the original NiCu LDH can be indexed as the (003), (006), (101), (012), (015), and (018) crystal faces of hexagonal hydrotalcite (PDF#09-0418). The characteristic peaks of metal Cu (PDF#04-0836, attributed to the (111) crystal faces of cubic Cu) appear on the XRD patterns of Cu_1.25_/NiCu LDH, Cu_2.5_/NiCu LDH, and Cu_5_/NiCu LDH. With the increase of Cu loading, the diffraction peaks of Cu significantly increase, which proves the successful synthesis of Cu/NiCu LDH nanostructures. Moreover, the characteristic peaks located around 467.7 and 517.5 cm^−1^ can be observed from the Raman spectrum (Figure S2), which belong to the stretching vibration mode of Ni^II^-O in Cu/NiCu LDH and NiCu LDH.

Scanning electron microscopy (SEM) image (Figure 1b and Figures S3–S6) shows that Cu/NiCu LDH has a similar nanosheet morphology to the original NiCu LDH, with Cu nanoparticles clearly attached to the surface. Transmission electron microscopy (TEM) analysis also showed that Cu/NiCu LDH samples showed the topography of nanosheets (Figure 1c) Cu nanoparticles (pointed by arrows) dispersed on NiCu LDH. Lattice spacing of 0.17 nm in high-resolution TEM (HRTEM) image (Figure 1d) can be assigned to (110) crystal plane of hexagonal NiCu LDH, and Cu nanoparticle with a size of about 15 nm presents a lattice spacing of 0.21 nm of (101) plane. The element mapping image illustrates the uniform distribution of Ni, Cu, and O in Cu/NiCu LDH (Figure 1e and Figures S3–S6), in addition, the specific ratio of Ni, Cu, and O are shown in Table S1.

**Figure 2:**
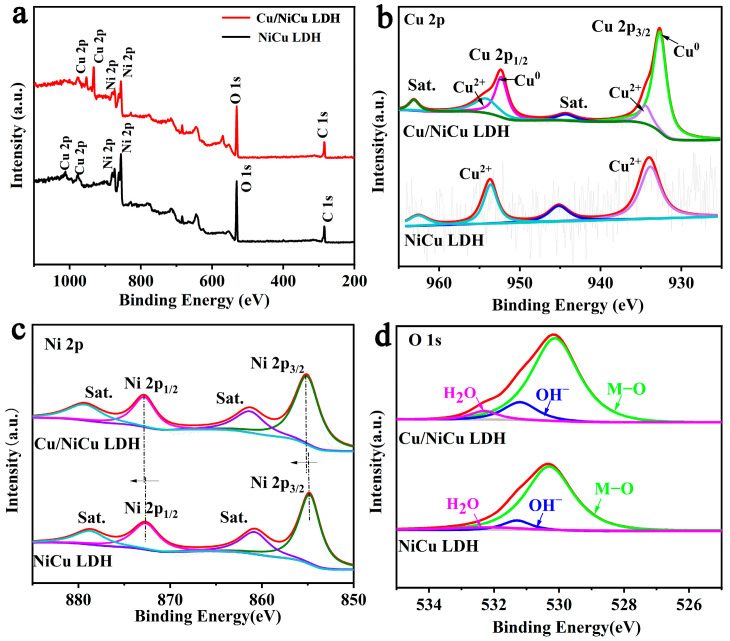
(**a**) survey, (**b**) Cu 2p, (**c**) Ni 2p, and (**d**) O 1s XPS spectra of Cu_2.5_/NiCu LDH and NiCu LDH.

As shown in Figure 2, the elemental composition and surface chemical state of Cu/NiCu LDH were confirmed by X-ray photoelectron spectroscopy (XPS) [31]. The XPS measurement spectrum (Figure 2a) confirmed the presence of Ni, Cu, and O elements in the prepared sample. In the spectrum of Cu 2p (Figure 2b), the 2p orbital deconvolution of NiCu LDH is Cu 2p_3/2_ and Cu 2p_1/2_. It is worth noting that Cu in NiCu LDH is mainly +2 valence, while Cu in Cu/NiCu LDH has a characteristic peak of Cu^0^ in addition to the characteristics of Cu^2+^, which proves the existence of Cu nanoparticles [32]. The Ni 2p signal (Figure 2c) can be deconvolved into two characteristic peaks. In addition to the two satellite peaks (Note Sat.), Cu/NiCu LDH and NiCu LDH also contain a pair of spin-orbiting peaks, Ni 2p_3/2_ and Ni 2p_1/2_, respectively. Compared with NiCu LDH, the peaks of Ni 2p_3/2_ (855.23 eV) and Ni 2p_1/2_ (872.97 eV) of Cu/NiCu LDH are positively shifted, which indicates that the introduction of Cu nanoparticles reduces the electron concentration around Ni and increases the oxidation valence of Ni [33]. According to previous studies, high-valent Ni both favors the formation of metal hydroxide active phases and optimizes the electronic structure of hydroxides to facilitate OER kinetics [34]. In the O 1s spectrum (Figure 2d), three distinct peaks are located at 530.8 eV, 531.6 eV, and 533.2 eV. The de-convoluted O 1s spectra can be indexed into three O species with different coordination environments: physically adsorbed water molecules, hydroxides, and metal-bonded oxygen species [35,36]. In conclusion, XPS results prove that the introduction of Cu nanoparticles can significantly regulate the electronic structure of NiCu LDH, proving that strong electron interaction occurs between Cu and NiCu LDH [37,38].

**Figure 3:**
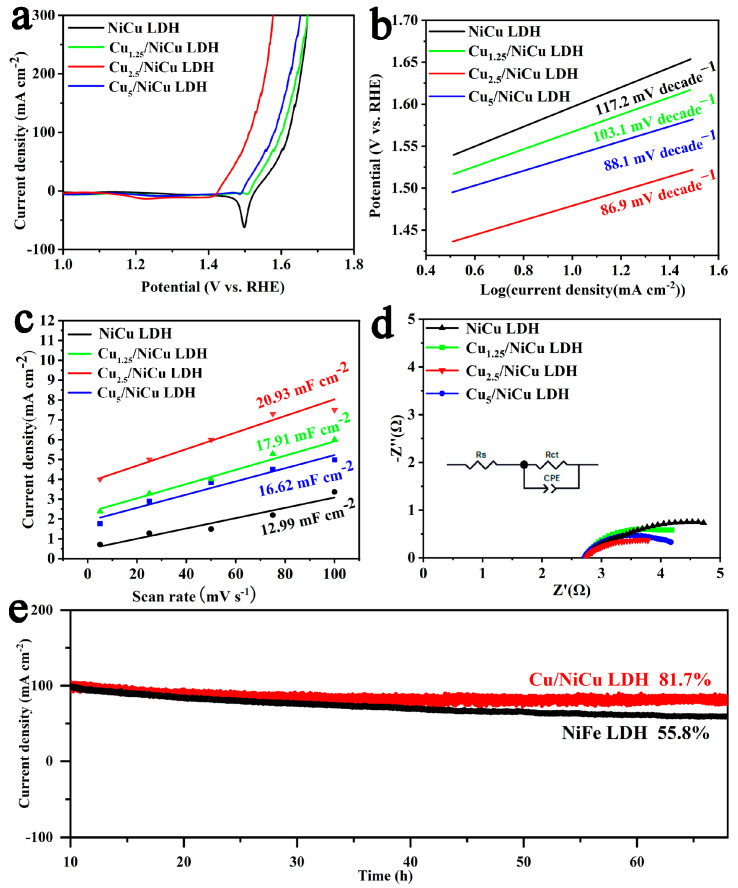
(**a**) LSV curves, (**b**) Tafel plots, (**c**) Cdl plots, and (**d**) Nyquist plots of the NiCu LDH Cu_1.25_/NiCu LDH, Cu_2.5_/NiCu LDH and Cu_5_/NiCu LDH for OER in 1 M KOH solution. (**e**) Long-term stability test of Cu_2.5_/NiCu LDH and NiFe LDH under the constant potential of 1.6 V for 70 h.

The OER catalytic performance of Cu_1.25_/NiCu LDH, Cu_2.5_/NiCu LDH, Cu_5_/NiCu LDH and NiCu LDH was evaluated in 1 M KOH using a three-electrode system. Linear scanning voltammetry (LSV) for all samples is shown in Figure 3a, Cu_2.5_/NiCu LDH requires only 206 mV overpotential to drive a current density of 10 mA cm^−2^, which is 100, 84, and 67 mV lower than NiCu LDH, Cu_1.25_/NiCu LDH, and Cu_5_/NiCu LDH. Besides, comparing with other advanced electrocatalysts, Cu_2.5_/NiCu LDH still behave outstanding performance (Table S2). These results show that the presence of a certain amount of Cu nanoparticles has a positive effect on improving the OER activity of NiCu LDH. In addition, the Tafel slope was used to evaluate the kinetics of Cu/NiCu LDH catalytic reactions [39]. As shown in Figure 3b, Cu_2.5_/NiCu LDH (86.9 mV dec^−1^) has the lowest Tafel slope compared to the Tafel slopes of NiCu LDH (117.2 mV dec^−1^), Cu_1.25_/NiCu LDH (103.1 mV dec^−1^), and Cu_5_/NiCu LDH (88.1 mV dec^−1^). As shown in Figure 3c, the electrochemically active surface area (ECSA) of the catalyst is determined by measuring the bilayer capacitance (C_dl_) due to the positive correlation linear relationship [40]. We used cyclic voltammetry (CV) to calculate the C_dl_ of catalysts in the experiments. As shown in Figure S7. Compared with the original NiCu LDH (12.99 mF cm^−2^), Cu_1.25_/NiCu LDH (16.62 mF cm^−2^) and Cu_5_/NiCu LDH (17.91 mF cm^−2^), Cu_2.5_/NiCu LDH had the highest C_dl_ value of 20.93 mF cm^−2^, indicating that Cu_2.5_/NiCu LDH had the largest catalytically active surface area (Table S3). More active sites are exposed and more electrocatalytic active centers are generated. In addition, the current density normalized by ECSA (Figure S8) showed that Cu_2.5_/NiCu LDH had better intrinsic activity than NiCu LDH, Cu_1.25_/NiCu LDH nd Cu_5_/NiCu LDH [41].To evaluate the charge transfer resistance at the solid-liquid interface, electrochemical impedance spectroscopy (EIS) was measured. The Nyquist plot is shown in Figure 3d. The Rct value (2.43 Ω) of Cu_2.5_/NiCu LDH catalyst is the smallest, indicating that the introduction of Cu promotes charge transfer between NiCu LDH and electrolyte [42]. In addition to electrocatalytic activity, the long-term stability of OER catalysts is also important for practical applications. As shown in Figure 3e, after the long-term stability test of Cu_2.5_/NiCu LDH at a constant overvoltage of 1.6 V lasted for 70 h, the OER current response showed only a slight attenuation. The best active NiFe LDH only maintained 55.8% of the activity within 70 h. In addition, the LSV curve measured after 3000 cycles of CV for Cu_2.5_/NiCu LDH has no significant current density decay compared to the initial LSV curve (Figure S9). It is proved that Cu/NiCu LDH not only has good activity, but also has excellent stability. Further, we characterized Cu/NiCu LDH after stability testing. XRD showed that Cu nanoparticles remained after stability testing, maintaining the heterogeneous structure well (Figure S10). XPS also shows that Cu^0^ still exists (Figure S11). SEM image shows that morphology did not change after test (Figure S12). Mapping shows that Ni, Cu, O elements is evenly distributed throughout the nanosheet region without segregation and aggregation (Figure S13). In addition, HRTEM showed that the surface of NiCu LDH produced an amorphous MOOH layer about 20 nm thick (Figure S14), which is a common phenomenon for OER catalysts, and this amorphous layer is considered the true active center of OER.

**Figure 4:**
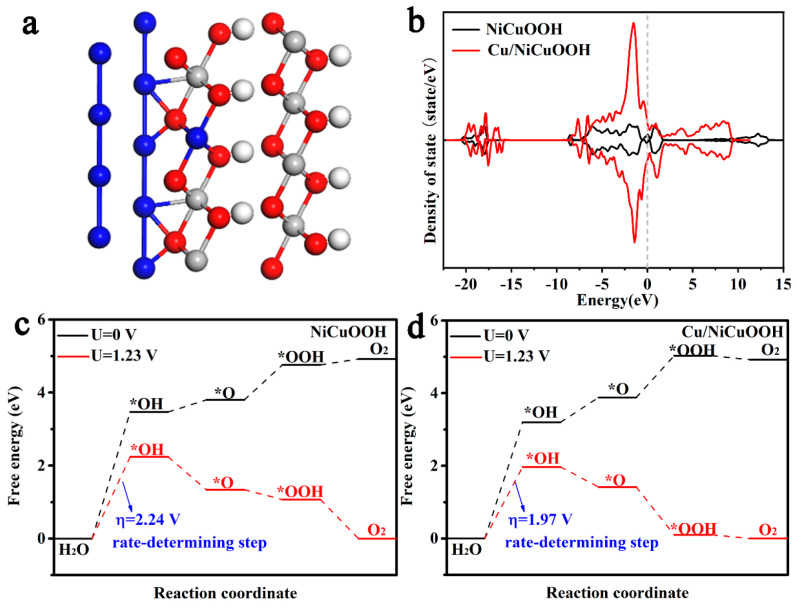
(**a**) The structure model of Cu/NiCuOOH (Cu: blue, Ni: gray, O: red, H: white). (**b**) Total density of states of NiCuOOH and Cu/NiCuOOH. Calculated free energy diagrams for the OER at different potentials of (**c**) NiCuOOH and (**d**) Cu/NiCuOOH.

To further reveal the influence of Cu nanoparticles on the electronic structure of NiCu LDH at the atomic scale, we performed density functional theory (DFT) calculations. It has been previously demonstrated that Cu/NiCu LDH is converted to Cu-loaded NiCu hydroxyoxide (Cu/NiCuOOH) during electrochemical testing. Therefore, Cu and NiCuOOH were selected to construct a heterogeneous structural model. According to the exposed crystal plane in the TEM, the (101) plane of Cu and (110) plane of NiCuOOH were selected as the coupling interface. The model diagram of Cu/NiCuOOH is shown in Figure 4a, and the NiCuOOH model (Figure S15) is constructed for comparison. Figure 4b shows the total density of states (DOS) of NiCuOOH and Cu/NiCuOOH, with both catalysts exhibiting metallic properties due to their non-zero density at Fermi energy, and Cu/NiCuOOH displaying more electron filling at the Fermi level than NiCuOOH, indicating better conductivity [43,44]. In addition, we also calculated the Gibbs free energy of NiCuOOH and Cu/NiCuOOH during the OER reaction, which contains three typical oxygen-containing intermediates (*OH, *O, and *OOH), with Ni being the active site [45,46]. As shown in Figure 4c, the rate determination step (RDS) on NiCuOOH is the first step (from H_2_O to *OH) with an overpotential of 2.24 V. After the introduction of Cu nanoparticles, the RDS of the OER reaction is still the first step from H_2_O to *OH, but its overpotential is significantly reduced to 1.97 V (Figure 4d). Therefore, the introduction of Cu nanoparticles can optimize the adsorption energy of intermediates by adjusting the electronic structure of the active site of NiCuOOH, thereby significantly improving the OER intrinsic activity of NiCuOOH.

## 4. Conclusions

In conclusion, we designed a Cu nanoparticle modulated NiCu LDH catalyst for the first time. The Cu/NiCu LDH catalyst exhibited a low over-potential of 206 mV and a low Tafel slope of 86.9 mV dec^−1^ at the current density of 10 mA cm^−2^ in 1.0 M KOH and displayed an excellent long-term stability. Experimental analysis and DFT calculations suggest that the enhanced OER activity is ascribed to the increased electrochemical active sites, accelerated interfacial charge transfer and optimized adsorption and desorption of OER intermediate species. What makes this work novel is the construction and resolution of a Cu-containing particle LDH heterostructure interface, which has never been reported before, providing insights into the fabrication of robust and durable OER electrocatalysts under alkaline conditions.

## Data Availability

The datasets used during the current study are available from the corresponding author upon reasonable request.

## Supplementary Materials

The following supporting information can be downloaded at: https://www.mdpi.com/article/10.3390/ma16093372/s1, References [47,48,49,50,51,52,53,54] are cited in the supplementary materials.
