# Supplementary material for: Engineering Cu/NiCu LDH Heterostructure Nanosheet Arrays for Highly-Efficient Water Oxidation
Source: Materials (Basel). 2023 Apr 25;16(9):3372. doi: 10.3390/ma16093372 (PMC10179677; doi:10.3390/ma16093372)
Supplement: Supplementary file 1 [file materials-16-03372-s001.zip › materials-2316873-supplementary.pdf]

Engineering Cu/NiCu LDH Heterostructure Nanosheet Arrays for Highly-Efficient Water Oxidation

Ao-Bing Wang [1], Xin Zhang [1], Hui-Juan Xu [1,*], Li-Jun Gao [1], Li Li [1], Rui Cao [1] and Qiu-Yan Hao [2,*]

1 Hebei Key Laboratory of Man-Machine Environmental Thermal Control Technology and Equipment, Hebei Vocational University of Technology and Engineering, Xingtai 054000, China
2 School of Materials Science and Engineering, Hebei University of Technology, Tianjin 300130, China
* Correspondence: xuhuijuan_xpc@163.com (H.-J.X.); haoqiuyan@hebut.edu.cn (Q.-Y.H.)

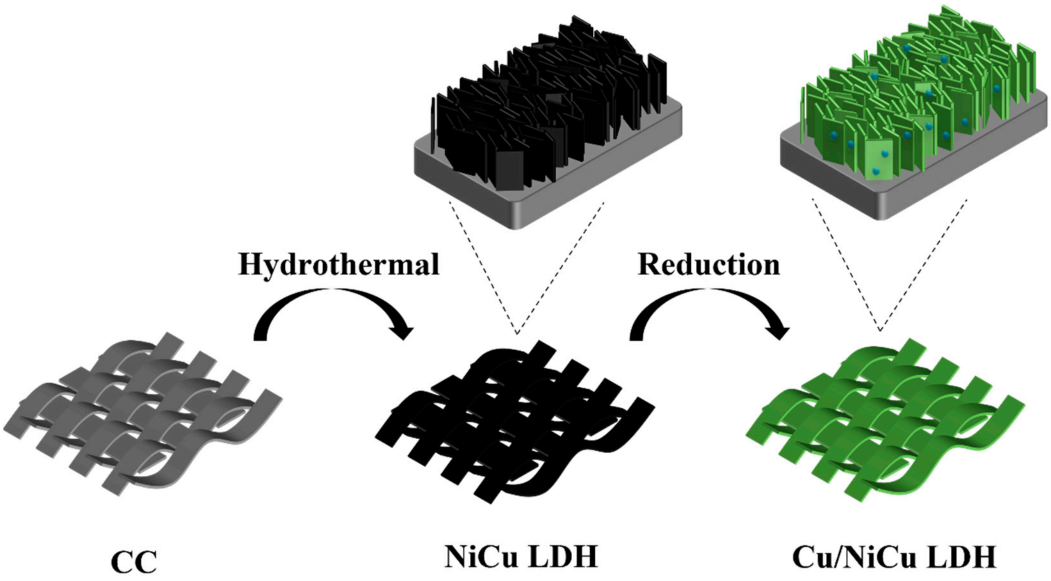

Figure S1. Schematic diagram of the preparation process of Cu/NiCu LDH.

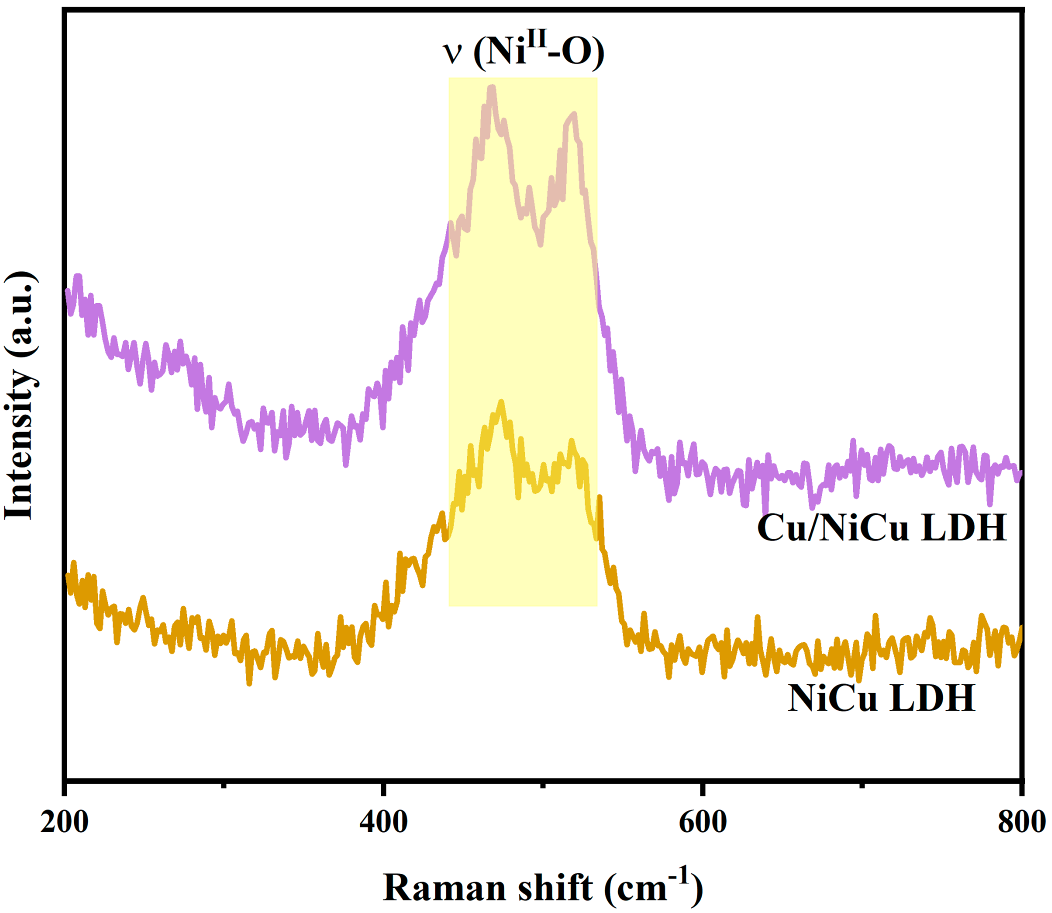

Figure S2. Raman spectrum of the synthesized NiCu LDH and Cu/NiCu LDH.

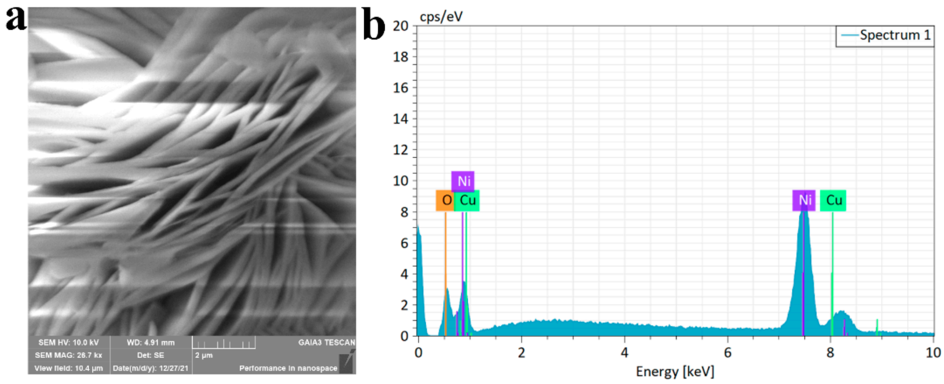

Figure S3. (a) SEM image and (b) EDS spectrum of NiCu LDH.

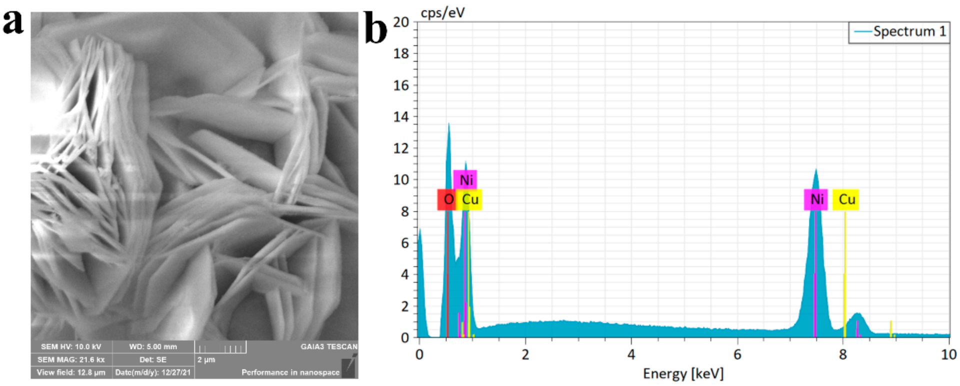

Figure S4. (a) SEM image and (b) EDS spectrum of $Cu_{1.25}$/NiCu LDH.

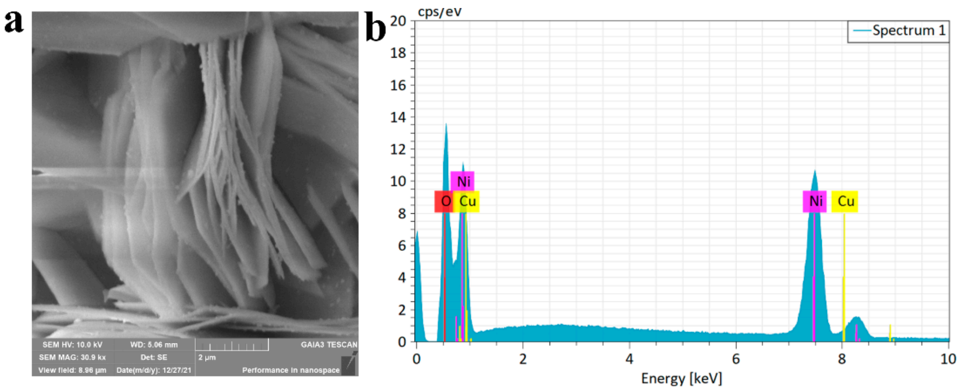

Figure S5. EDS spectrum of $Cu_{2.5}$/NiCu LDH.

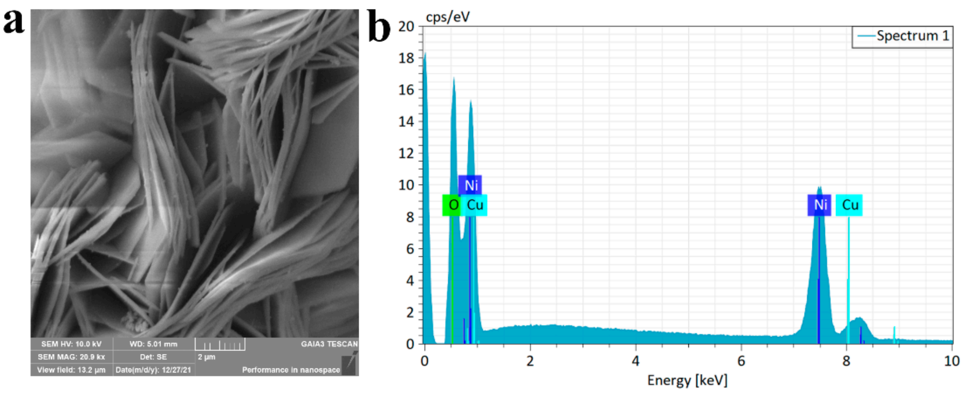

Figure S6. (a) SEM image and (b) EDS spectrum of Cu_5/NiCu LDH.

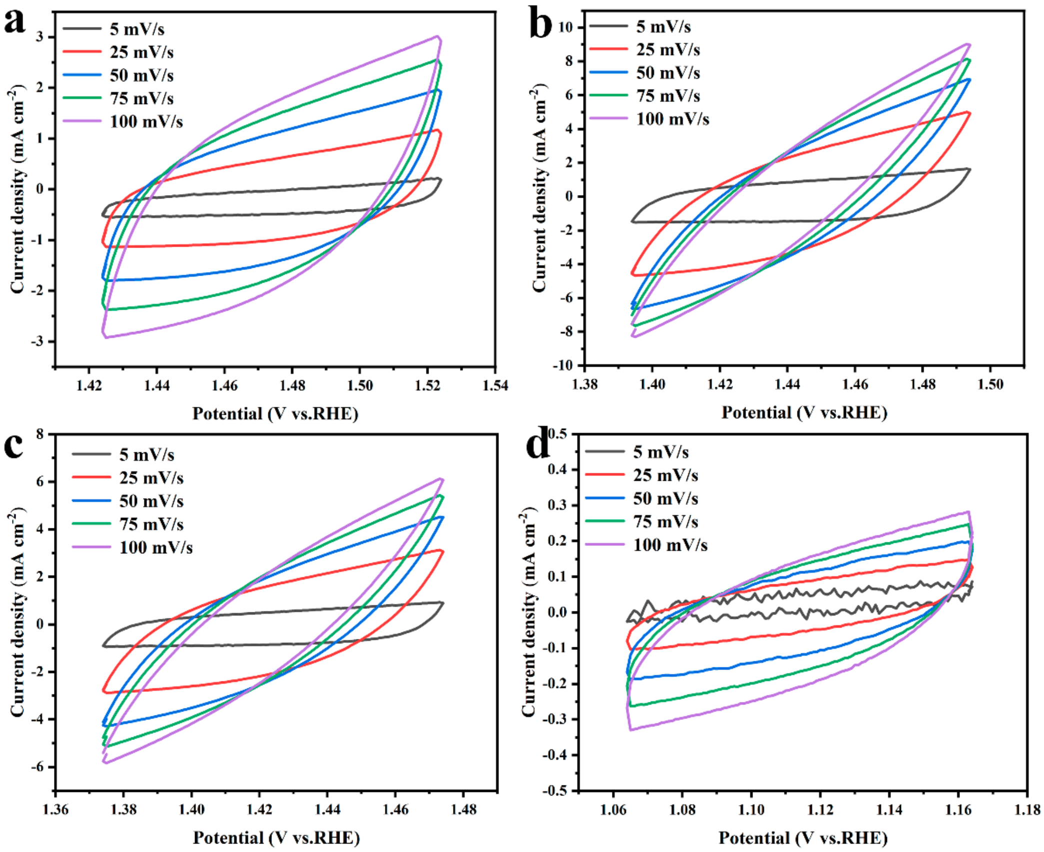

Figure S7. CV curves of (a) NiCu LDH, (b) $Cu_{1.25}$/NiCu LDH, (c) $Cu_{2.5}$/NiCu LDH and (d) Cu_5/NiCu LDH.

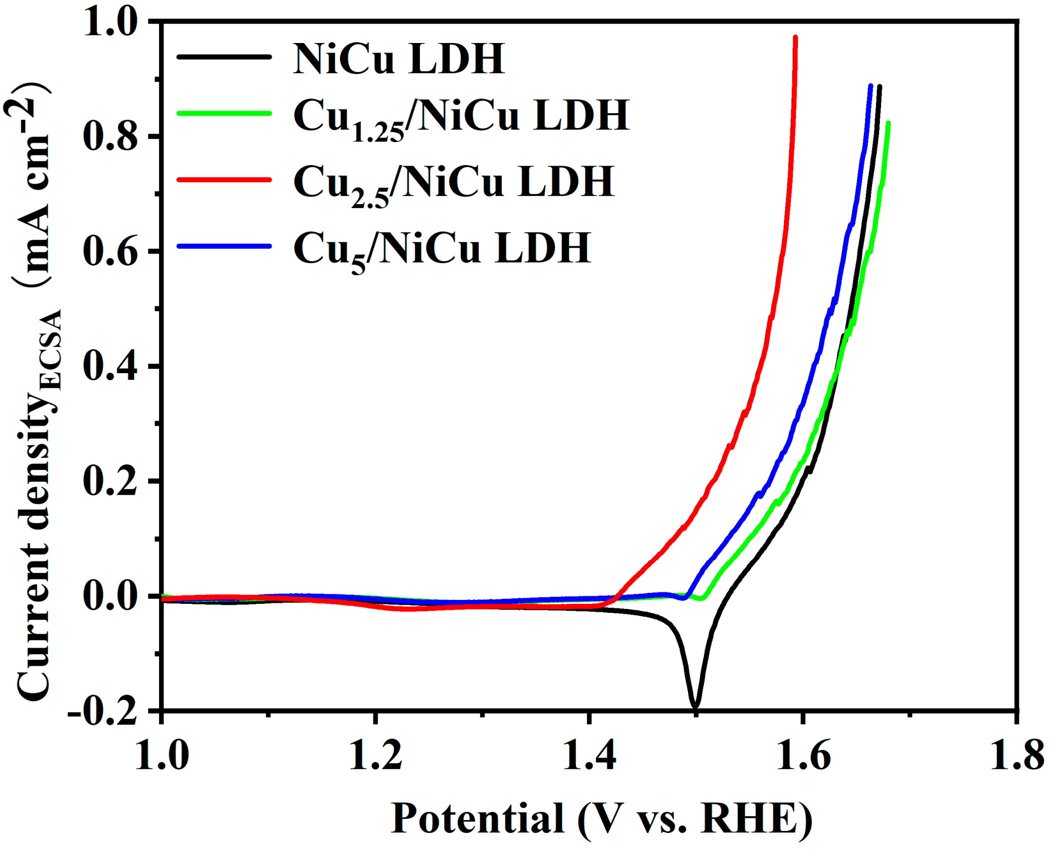

Figure S8. Normalized LSV curves using the ECSA value of Cu/NiCu LDH, $Cu_{1.25}$/NiCu LDH, $Cu_{2.5}$/NiCu LDH and Cu_5/NiCu LDH.

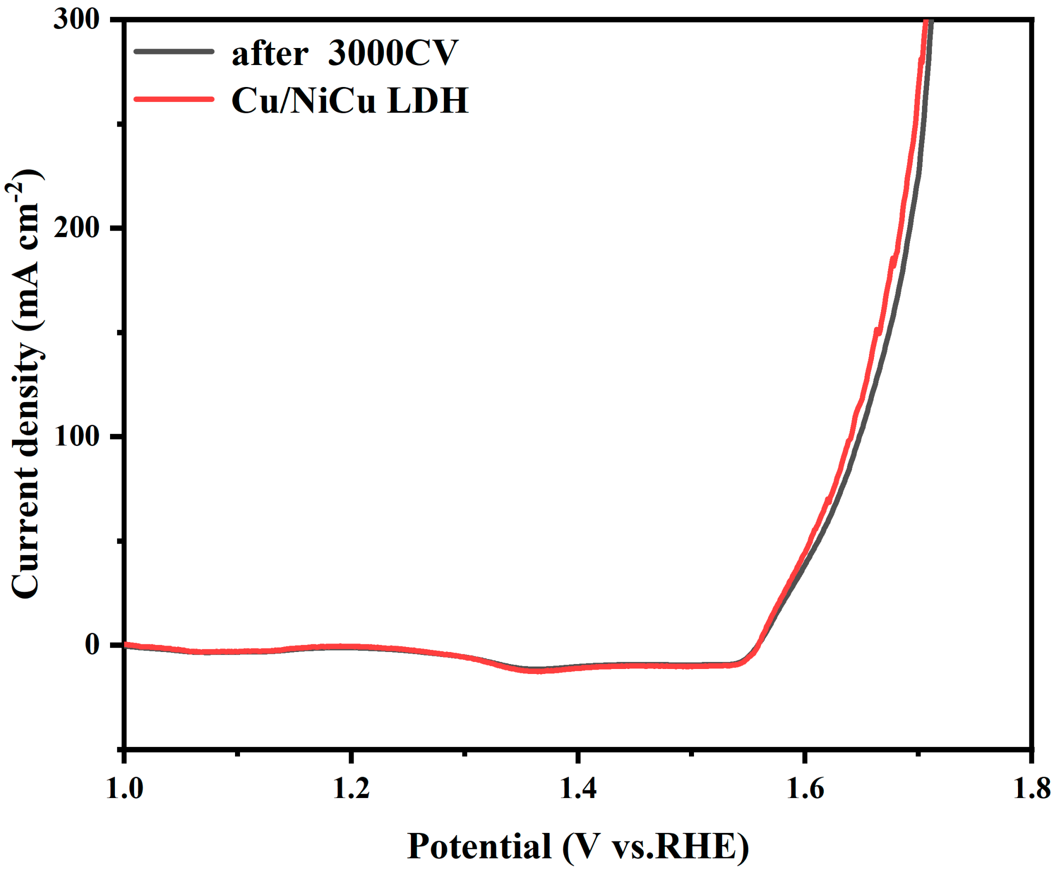

Figure S9. The LSV curves of Cu/NiCu LDH before and after 3000 CV tests.

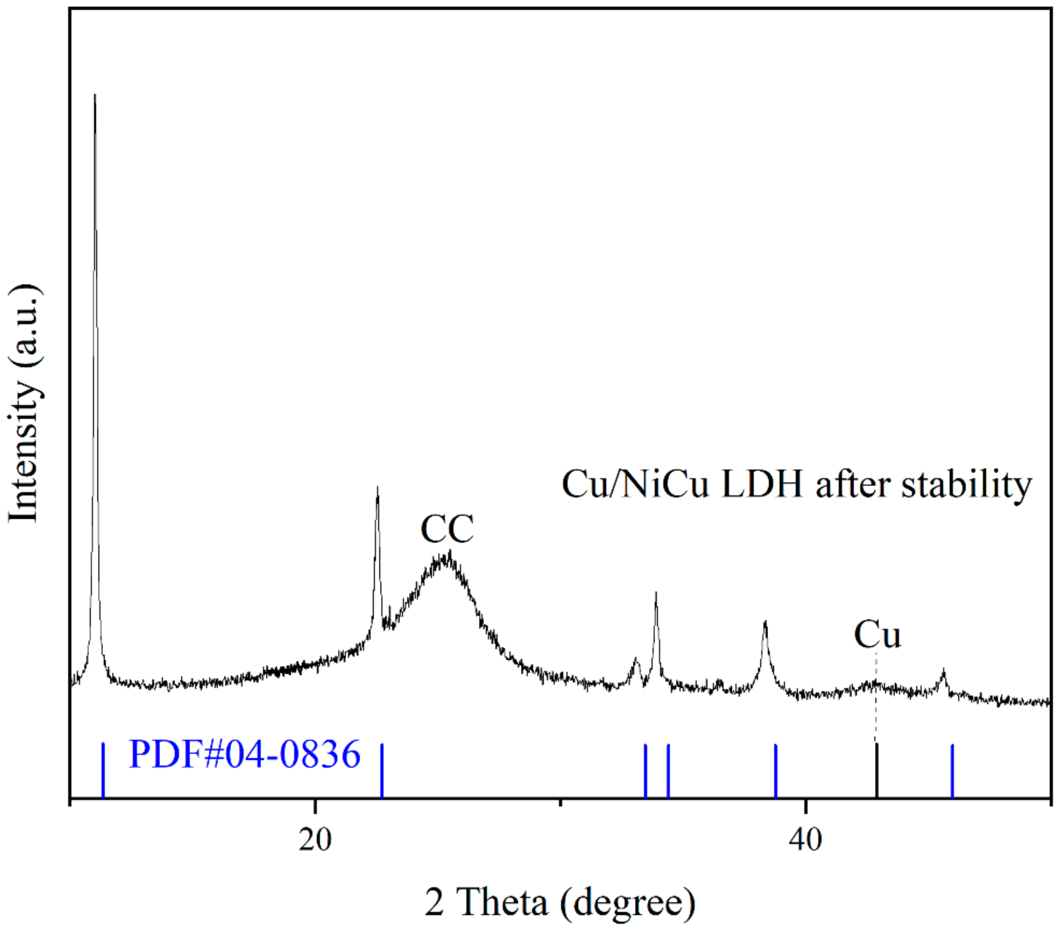

Figure S10. XRD pattern of $Cu_{2.5}$/NiCu LDH after stability test.

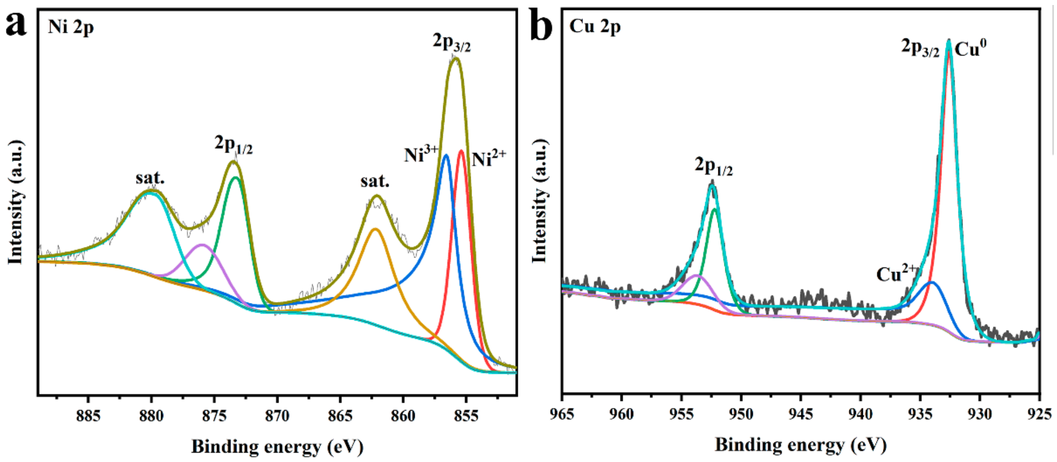

Figure S11. (a) Ni 2p and (b) Cu 2p XPS spectra of $Cu_{2.5}$/NiCu LDH after stability.

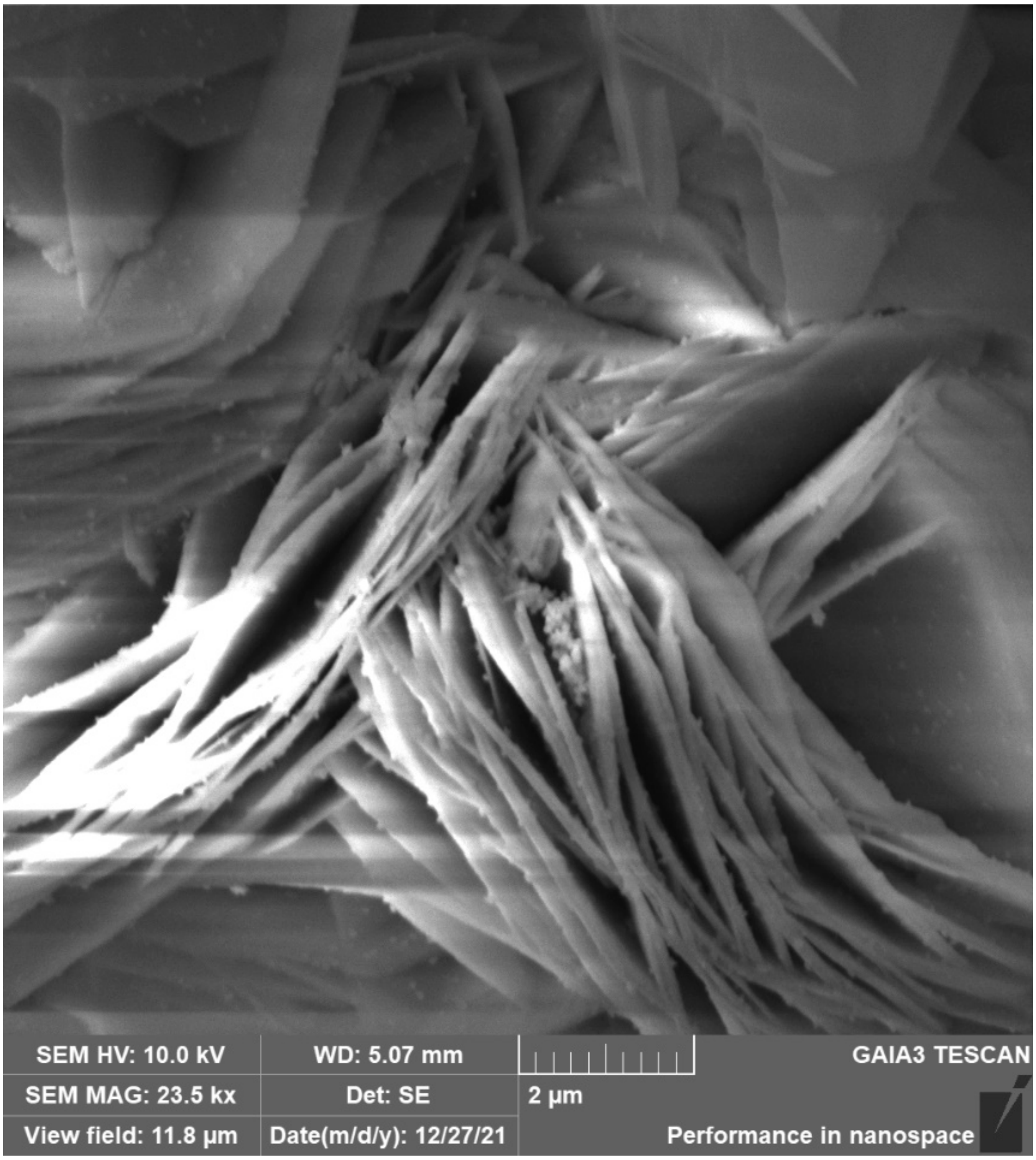

Figure S12. SEM image of $Cu_{2.5}$/NiCu LDH after stability test.

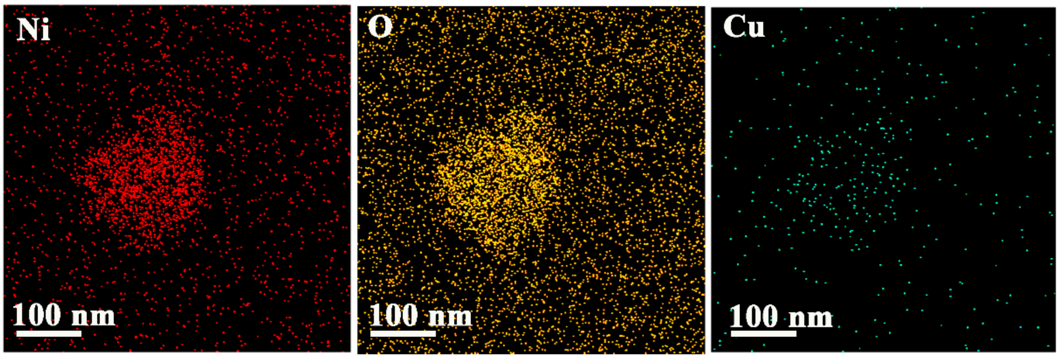

Figure S13. Element mapping of $Cu_{2.5}$/NiCu LDH after stability test.

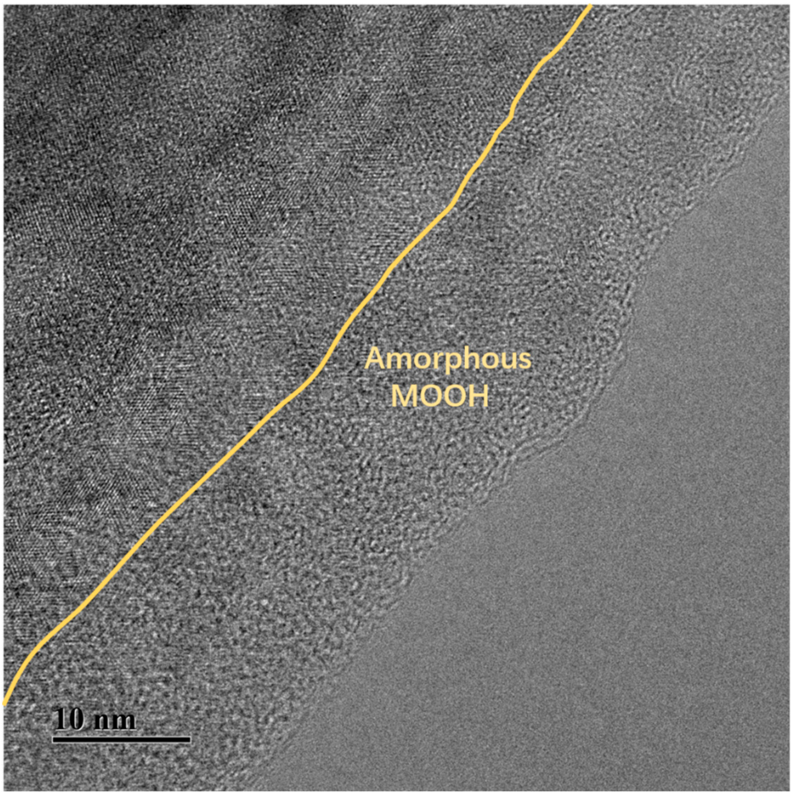

Figure S14. HRTEM image of $Cu_{2.5}$/NiCu LDH after stability test.

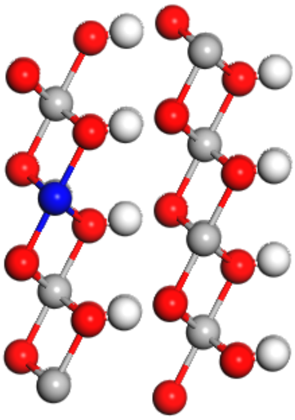

Figure S15. Constructed model of NiCuOOH.

Table S1. EDS results of NiCu LDH, $Cu_{1.25}$/NiCu LDH, $Cu_{2.5}$/NiCu LDH and Cu_5/NiCu LDH.

	Ni (at%)	Cu(at%)	O (at%)
NiCu LDH	34.58	1.08	64.34
$Cu_{1.25}$/NiCu LDH	25.94	1.95	72.10

$Cu_{2.5}$/NiCu LDH	27.86	2.52	69.62
Cu_5/NiCu LDH	30.34	6.14	63.52
$Cu_{2.5}$/NiCu LDH after stability test	20.7	2.31	76.99

Table S2. Comparison of OER performances of $Cu_{2.5}$/NiCu LDH with previously reported well-performed OER electrocatalysts.

Catalysts	η@10 mA cm^{-2} (mV)	Electrolyte	Substrate	Ref.
$Cu_{2.5}$/NiCu LDH	206	1M KOH	CC	This work
Mxene/NiFe LDH	260	1M KOH	RDE	[1]
GDY/NiFe LDH	260	1M KOH	CF	[2]
Pt/NiFe LDH	230	1M KOH	CC	[3]
Ni_2Cr_1 LDH/NF	319	1M KOH	NF	[4]
S–NiFe LDH/CC	206	1M KOH	CC	[5]
Ni/LDH-ZnO	210	1M KOH	NF	[6]
Exfoliated NiFe LDH	302	1M KOH	GC	[7]
NiO/NiFe LDH	270	1M KOH	GC	[8]
Fe–Ni LDH/MOFs	255	1M KOH	GC	[9]

Glassy carbon (GC); Ni foam (NF); carbon cloth (CC); Cu foam (CF); rotating disk electrode (RDE).

Table S3. ECSA values of NiCu LDH, $Cu_{1.25}$/NiCu LDH, $Cu_{2.5}$/NiCu LDH and Cu_5/NiCu LDH.

	NiCu LDH	$Cu_{1.25}$/NiCu LDH	$Cu_{2.5}$/NiCu LDH	Cu_5/NiCu LDH
ECSA values	324.75	447.75	523.25	415.5

Reference

1. Yu, M.; Zhou, S.; Wang, Z.; Zhao, J.; Qiu, J., Boosting electrocatalytic oxygen evolution by synergistically coupling layered double hydroxide with MXene. *Nano Energy* **2018,** 44, 181-190.
2. Shi, G.; Yu, C.; Fan, Z.; Li, J.; Yuan, M., Graphdiyne-Supported NiFe Layered Double Hydroxide Nanosheets as Functional Electrocatalysts for Oxygen Evolution. *ACS Appl. Mater. Inter.* **2019,** 11, 2662-2669.
3. Anantharaj, S.; Karthick, K.; Venkatesh, M.; Simha, T. V. S. V.; Salunke, A. S.; Ma, L.; Liang, H.; Kundu, S., Enhancing electrocatalytic total water splitting at few layer Pt-NiFe layered double hydroxide interfaces. *Nano Energy* **2017,** 39, 30-43.
4. Ye, W.; Fang, X.; Chen, X.; Yan, D., A three-dimensional nickel-chromium layered double hydroxide micro/nanosheet array as an efficient and stable bifunctional electrocatalyst for overall water splitting. *Nanoscale* **2018,** 10, 19484-19491.
5. Cao, L.-M.; Wang, J.-W.; Zhong, D.-C.; Lu, T.-B., Template-directed synthesis of sulphur doped NiCoFe layered double hydroxide porous nanosheets with enhanced electrocatalytic activity for the oxygen evolution reaction. *J. Mater. Chem. A* **2018,** 6, 3224-3230.
6. Luo, Y.; Wu, Y.; Wu, D.; Huang, C.; Xiao, D.; Chen, H.; Zheng, S.; Chu, P. K., NiFe-Layered Double Hydroxide Synchronously Activated by Heterojunctions and Vacancies for the Oxygen Evolution Reaction. *ACS Appl. Mater. Inter.* **2020,** 12, 42850-42858.
7. Song, F.; Hu, X., Exfoliation of layered double hydroxides for enhanced oxygen evolution catalysis. *Nat. Commun.* **2014,** 5, 4477.
8. Gao, Z.-W.; Liu, J.-Y.; Chen, X.-M.; Zheng, X.-L.; Mao, J.; Liu, H.; Ma, T.; Li, L.; Wang, W.-C.; Du, X.-W., Engineering NiO/NiFe LDH Intersection to Bypass Scaling Relationship for Oxygen Evolution Reaction via Dynamic Tridimensional Adsorption of Intermediates. *Adv. Mater.* **2019,** 31, 1804769.

9. Huo, J.; Wang, Y.; Yan, L.; Xue, Y.; Li, S.; Hu, M.; Jiang, Y.; Zhai, Q.-G., In situsemi-transformation from heterometallic MOFs to Fe-Ni LDH/MOF hierarchical architectures for boosted oxygen evolution reaction. *Nanoscale* **2020,** 12,14514-14523.
